# Individual differences in song plasticity in response to social stimuli and singing position

**DOI:** 10.1002/ece3.8883

**Published:** 2022-05-02

**Authors:** Mónika Jablonszky, David Canal, Gergely Hegyi, Katalin Krenhardt, Miklós Laczi, Gábor Markó, Gergely Nagy, Balázs Rosivall, Eszter Szász, Sándor Zsebők, László Zsolt Garamszegi

**Affiliations:** ^1^ Centre for Ecological Research Institute of Ecology and Botany Vácrátót Hungary; ^2^ Behavioural Ecology Group Department of Systematic Zoology and Ecology ELTE Eötvös Loránd University Budapest Hungary; ^3^ The Barn Owl Foundation Orosztony Hungary; ^4^ 72402 Department of Plant Pathology Institute of Plant Protection Hungarian University of Agriculture and Life Sciences Budapest Hungary; ^5^ MTA‐ELTE Theoretical Biology and Evolutionary Ecology Research Group Institute of Physics ELTE Eötvös Loránd University Budapest Hungary

**Keywords:** acoustic communication, behavioral variance, consistency, *Ficedula albicollis*, flexibility, SNPs

## Abstract

Individual animals can react to the changes in their environment by exhibiting behaviors in an individual‐specific way leading to individual differences in phenotypic plasticity. However, the effect of multiple environmental factors on multiple traits is rarely tested. Such a complex approach is necessary to assess the generality of plasticity and to understand how among‐individual differences in the ability to adapt to changing environments evolve. This study examined whether individuals adjust different song traits to varying environmental conditions in the collared flycatcher (*Ficedula albicollis*), a passerine with complex song. We also aimed to reveal among‐individual differences in behavioral responses by testing whether individual differences in plasticity were repeatable. The presence of general plasticity across traits and/or contexts was also tested. To assess plasticity, we documented (1) short‐scale temporal changes in song traits in different social contexts (after exposition to male stimulus, female stimulus or without stimuli), and (2) changes concerning the height from where the bird sang (singing position), used as a proxy of predation risk and acoustic transmission conditions. We found population‐level relationships between singing position and both song length (SL) and complexity, as well as social context‐dependent temporal changes in SL and maximum frequency (MF). We found among‐individual differences in plasticity of SL and MF along both the temporal and positional gradients. These among‐individual differences in plasticity were repeatable. Some of the plastic responses correlated across different song traits and environmental gradients. Overall, our results show that the plasticity of bird song (1) depends on the social context, (2) exists along different environmental gradients, and (3) there is evidence for trade‐offs between the responses of different traits to different environmental variables. Our results highlight the need to consider individual differences and to investigate multiple traits along multiple environmental axes when studying behavioral plasticity.

## Graphical abstract

This study examined in the collared flycatcher (*Ficedula albicollis*), whether individuals adjust different song traits to varying environmental conditions. We recorded a population‐level relationship between singing position and song length (SL), and repeatable among‐individual differences in plasticity of SL and maximum frequency along both the temporal (order) and positional (singing position) gradients. Our results show that the plasticity of bird song exists along different environmental gradients and that it may potentially evolve.

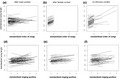

## 1 INTRODUCTION

Behavioral traits are inherently plastic, which has a considerable impact on the adaptation to environmental changes and on the evolution of behavioral traits (Charmantier & Gienapp, 2014; Snell‐Rood, 2013). However, plasticity has limits, for example, due to cognitive costs or the unreliability of environmental cues (Dewitt et al., 1998). Several studies have shown that the extent to which animals alter their behavior may vary among individuals (Mathot et al., 2011, 2012; Wolf et al., 2008) and that behavioral plasticity may be repeatable (Araya‐Ajoy & Dingemanse, 2017; Araya‐Ajoy et al., 2015). Adaptive differences in behavioral plasticity may emerge from several mechanisms (Dingemanse & Wolf, 2013), such as state differences among individuals (Houston & McNamara, 1999; Mathot et al., 2011; Nicolaus et al., 2012), temporal or spatial variation in resources in the presence of competition (Mathot et al., 2011; Wolf et al., 2008), or due to direct social interactions, which result in the emergence of socially responsive and unresponsive individuals (Dall et al., 2004; Wolf et al., 2011). Individual differences in plasticity may arise from either additive genetic background, different experiences or early environmental (e.g., maternal) effects (Araya‐Ajoy & Dingemanse, 2017; Dingemanse et al., 2012; Dingemanse & Wolf, 2013; Nussey et al., 2007). Therefore, behavioral plasticity could evolve as an individual‐specific trait subjected to selection through differential survival, mate choice, or reproductive success (Barou‐Dagues et al., 2020; Han & Brooks, 2013).

Animals are exposed to multiple environmental effects that may interactively affect their behavior (Dosmann & Mateo, 2014; Westneat et al., 2009, 2011). In addition, multiple behaviors may have similar or different responses to the same environmental factor (Araya‐Ajoy & Dingemanse, 2017; Dosmann et al., 2015; Moldoff & Westneat, 2017). Rigorous testing of multidimensional plasticity has rarely been carried out, even though such plasticity is probably common and it may have important effects on adaptation when individuals face environmental changes (Visser, 2008).

Bird song is an extremely variable behavioral trait shaped by sexual selection (Catchpole & Slater, 2008; Eriksson & Wallin, 1986) that plays an essential role in intra‐ and intersexual communication (Qvarnström et al., 2010; Vehrencamp et al., 2014; Warrington et al., 2014). Song is considered an honest signal because it is associated with various costs such as energy loss and increased predation risk (Gil & Gahr, 2002). Bird songs show a high variability at the among‐individual and within‐individual levels (Naguib et al., 2016; Palmero et al., 2012; Zsebők et al., 2017). Important factors that may drive plastic changes in song are the social environment (as a song is usually directed to conspecifics) and the singing position (by influencing signal transmission and predation risk; Dabelsteen et al., 1993; Götmark & Post, 1996). These factors may elicit different responses in different song traits (Bueno‐Enciso et al., 2016; Opaev & Kolesnikova, 2019). Additionally, song plasticity can be observed at various timescales, depending on the considered environmental driver. For example, population density may drive plastic responses among years, if individuals adjust song output to the current level of competition, whereas the composition of listener conspecifics may influence song on a shorter time scale (Gersick & White, 2018; Kipper et al., 2015; Patricelli & Krakauer, 2010). Bird song thus provides an excellent model to study plasticity.

The social environment changes continuously in highly mobile songbirds, so it may be advantageous to adjust singing behavior rapidly based on the current social audience (Patricelli & Krakauer, 2010). For example, males may sing differently depending on the sex and quality of the listeners (Heinig et al., 2014; Jablonszky et al., 2021; Ronald et al., 2015). Males should benefit from adjusting their investment in song according to the quality of the courted female, and in signals of aggressive intent according to the threat represented by the opponents (Fitzpatrick & Servedio, 2018; Maynard, 1982; Moser‐Purdy & Mennill, 2016).

Nonsocial environmental conditions can also affect song displays in the short term. For example, as signaling behavior is closely associated with predation risk (Gil & Gahr, 2002; Krams, 2001), it may be adjusted to an individual's level of exposure. Predator types may be different and predation pressure may be lower when birds sing concealed within the vegetation versus in more exposed sites (Götmark & Post, 1996; Møller, 2011a, 2011b; Møller et al., 2006). In addition, the physical environment may affect the properties of sound transmission (e.g., high‐frequency sounds rapidly degrade due to absorption by dense foliage; Barker et al., 2009; Dabelsteen et al., 1993). Thus, singers may adjust their songs according to the expected level of song degradation (Bueno‐Enciso et al., 2016; Mathevon et al., 1996; Nemeth et al., 2001). In temperate forests, the height of the singing position can be a good proxy for the exposure of the birds as the foliage becomes denser higher in the canopy and, therefore, it could be associated with both predation risk and song degradation. However, the plastic response of bird song to the vertical singing position remains unexplored.

Here, we aimed to investigate whether individuals plastically adjust multiple characteristics of their songs along multiple environmental gradients and whether individuals differ in such adjustments, using a passerine bird with complex song, the collared flycatcher (*Ficedula albicollis*). We evaluated whether plasticity covaries between traits and between environmental gradients at the individual level. We also examined if individual differences in song plasticity were repeatable. To assess song plasticity, we documented short‐term variations in different song traits associated with changes in both social and nonsocial environments using the reaction norm approach (Dingemanse et al., 2010). To assess the effect of the social environment, we exposed individuals to different social contexts before recording the song and observed the effect of social context over time within the recording. We expected that males react to the change in the social context by gradually changing their song after the disappearance of a potential mate or rival. To estimate the effect of a nonsocial environmental factor, we used the height of the singing position as a proxy, assuming that higher singing positions would represent more concealed acoustic habitats than lower positions.

We predicted that if there are individual differences in the costs and benefits of energy investment in a specific social context, males might respond to the changing social context with individually different plasticity. As song traits may play different roles in communication, plastic response to social context may also differ among them. Degradation effects at a given singing position may be expected to manifest similarly across individuals. If so, and if plastic responses in song to the singing position primarily reflect song degradation, this should result in common patterns in song plasticity across individuals. However, individual variation in plastic response in song to singing position height is expected if song adjustments to the singing positions are related to constraints associated with song production costs, or if they reflect individual risk‐taking tendencies (Garamszegi et al., 2008).

## 2 METHODS

### 2.1 Study site and model species

The study was conducted in an oak‐dominated forest in the Pilis‐Visegrádi Mountains, Duna‐Ipoly National Park, Hungary (47°43'N, 19°01'E). The study plot contains approximately 800 artificial nest boxes.

The collared flycatcher is a hole‐nesting, long‐distance migratory passerine. Most males arrive earlier (around the middle of April) than females to the breeding grounds and occupy territories that consist of a small area around a natural tree cavity or a nest box. Male collared flycatchers start singing after arriving and usually stop singing after pairing (Garamszegi et al., 2004; Pärt, 1991).

The territorial song performance of males, used to attract potential mates and dissuade rivals, consists of sequences of 3–5‐second‐long songs composed of syllables (the smallest song unit), separated from each other by a few sec intervals (Gelter, 1987). Collared flycatcher males have a repertoire size of 20–100 syllables estimated from 20 songs per individual (Garamszegi et al., 2006; Zsebők, Herczeg, et al., 2018). Song is correlated with certain aspects of individual quality (Garamszegi et al., 2003, 2006, 2007) and is associated with mating success and male–male competition (Garamszegi et al., 2004; Hegyi et al., 2010). In addition, the height of the singing position is negatively associated with pairing speed (Garamszegi et al., 2008). Repeatability estimates of song traits revealed that within‐individual variance is considerable even within a short time period (Zsebők et al., 2017). This variance is not stochastic, but may be of biological relevance, as male collared flycatchers seem to alter their song according to the identity of listeners (Jablonszky et al., 2021).

### 2.2 Field procedures

Data were collected during the courtship period of the collared flycatcher (within the period from 11 April to 7 May) between 2008 and 2019.

First, we captured males and females to be used as stimulus birds to elicit songs from the focal males (see details at Jablonszky et al., 2021). Stimulus birds were captured in different study plots (located at least 500 m away) than the tested males. Before starting the song recordings, the stimulus birds were placed into small cages (15 × 20 × 15 cm), with food (mealworms) provided ad libitum. We usually used the same stimulus bird for multiple song recordings (mean = 4.9 ± 4.0) to reduce the number of stimulus birds required during the assays (see below). The use of live birds as stimuli was necessary as males reacted differently to stuffed decoys (personal observations of the authors).

We monitored the study area daily for newly arrived, unpaired birds, displaying near their occupied nest boxes. After finding a displaying male, we presented them with either (1) a male decoy as a social stimulus, mimicking natural situations associated with territorial intrusion or (2) a female stimulus, mimicking nest box inspection by females during mate‐sampling. The displayed stimulus was chosen based on field constraints (such as the availability of stimulus birds and the capacity of recording experimenters). During the assays, stimulus females were placed on the top of the nest box, while stimulus males were positioned 1.5–2.0 m away from the nest box. The different positions were necessary to represent better the different natural situations mimicked with the stimuli. The focal males usually stopped singing and fled away when we approached the nest box. They were exposed to the stimuli for 5 min after returning to their territory. This time period has been reported to be sufficient for the focal male to interpret the situation as a visit from a prospecting female or a territorial intrusion (Garamszegi et al., 2008). We measured other behavioral traits of the focal birds during exposure, when they typically did not sing. These behavioral traits were independent from song traits (Garamszegi et al., 2008), and thus were not included in the present analyses. To minimize disturbance, we presented a stimulus and recorded song for a focal male typically only once on a given day.

After removing the stimulus, we recorded the song of the focal males using a standard protocol (Garamszegi et al., 2006; Zsebők, Herczeg, et al., 2018). We used a Telinga parabola dish with a Sennheiser ME62 microphone and K6 preamplifier on Tascam DR1 and Microtrack II handheld digital recorders (48 kHz sampling rate and 16‐bit quality). If the focal bird was visible during the recording, we documented the singing position. The singing position was defined as the position of the bird relative to the height of the vegetation (the average height of trees in our study area is around 25 m) in percentage, varying from 0% (ground) to 100% (top of the canopy). We used relative singing position instead of absolute singing position, as the relative singing position adequately describes the differences in concealment of the singing birds in the vegetation due to the similar habitats where the songs of the birds were recorded. Recordings were performed only in the absence of rain and strong wind, lasted at least 10 min and included at least 20 songs (Zsebők et al., 2017). Songs with major disturbance from other birds, such as direct contact with conspecifics, were not used in the further analyses. Due to logistic constraints, approximately one quarter of the song recordings was made without presenting a stimulus bird. Otherwise, the same recording protocol was used. These recordings could not serve as a control to the songs recorded after the presentation of a stimulus, as the social context could not be controlled. However, they can be considered as a different social situation where the birds did not sing to an immediate conspecific. Thus, we included this category in the analysis to reflect a different background situation for the social context.

We captured the focal males within an hour after the song recordings for ringing and to obtain morphological measurements. Birds without rings were marked with individually numbered rings (Aranea, Poland). We determined the age of males (i.e., 1‐year old or adult) based on their plumage (Mullarney et al., 1999). All applicable international, national, and institutional guidelines for the care and use of animals were followed. Permissions for the fieldwork have been provided by the Middle‐Danube‐Valley Inspectorate for Environmental Protection, Nature Conservation and Water Management, ref. nos.: KTVF 16360‐2/2007, KTVF 30871‐1/2008, KTVF 43355‐1/2008, KTVF 45116‐2/2011, KTVF 21664‐3/2011, KTVF 12677‐4/2012, KTVF 10949‐8/2013, KTF 11978‐5/2015, PEI/001/1053‐6/2015, PE/EA/101‐8/2018, PE‐06/KTF/8550‐4/2018, and PE‐06/KTF/8550‐5/2018) and the work was also approved by the ethical committee of the Eötvös Loránd University (ref. no. TTK/2203/3).

### 2.3 Analysis of song recordings

Overall, we analyzed songs from 185 males (from 9 to 126 songs per record). A total of 98 recordings (3,434 songs) were made for 85 males, after presenting male stimuli, 46 recordings (1092 songs) were made for 45 males after presenting female stimuli and 73 recordings (2046 songs) were recorded for 66 males without a stimulus.

Three independent song traits with biological relevance in the study population were used during the statistical analysis. Specifically, we considered for each song the song length (SL), maximum frequency (MF), and short‐term complexity (hereafter complexity), which probably play a role in inter‐ or intrasexual communication in the study species (Jablonszky et al., 2021) and has been found to be associated with singing position height (Azar & Bell, 2016; Mathevon et al., 1996; Nemeth et al., 2001). We also extracted the corresponding singing position estimates whenever possible.

We manually cut out the songs from the recordings using the Adobe Audition 3.0 (Adobe Systems) software. We chose only songs for which the spectrograms were clearly distinguishable from the background noise. Syllables were manually selected and from the syllable segments we automatically extracted five spectrographic features with the Ficedula Toolbox (Zsebők, Blázi, et al., 2018): duration, minimum and MF, frequency bandwidth, and mean frequency of the syllable. The last variable was calculated as the mean of the peak frequency values in each spectrographic time window at the syllable level (Garamszegi et al., 2012). We grouped the syllables into 200 syllable types based on the five acoustic variables measured using the *k*‐means method in R (‘kmeans’ function in the ‘vegan’ R package; Oksanen et al., 2016; Zsebők, Blázi, et al., 2018).

At the song level, we measured SL and complexity. The latter was calculated as the number of different syllable types divided by the total number of syllables within songs. Additionally, we calculated the MF of the song as the maximum mean frequency value of the syllables within the song.

### 2.4 Statistical analyses

#### 2.4.1 Among‐individual (co)variance in song plasticity

We investigated the presence of among‐individual variation in song plasticity in short song recordings using linear mixed models (LMMs), applying the reaction norm approach (Dingemanse et al., 2010). We examined changes in the focal song traits in response to the order of songs (as songs recorded right after the presence of the stimulus can be expected to show stronger social influence than songs produced long after the removal of the stimulus). We also examined whether focal song traits varied along with the singing position, which we used as a proxy for environmental factors that vary along the vertical scale from the ground to the top of the canopy (i.e., sound degradation, predation risk). Specifically, we estimated among‐individual variation in deviations from the population slope (or individual × environment interaction) for the order of songs and singing position by including random slopes into multivariate random regression models (see below). We estimated linear reaction norms, as we predicted linear changes both after a change in the social context and according to the height of the singing position (Götmark & Post, 1996; Møller et al., 2006). Using these models, we could also assess the covariation between random intercept and slopes and between the random slopes within the traits, and between the random intercepts and slopes of the different behavioral traits. A correlation between a random intercept and slope within the traits would suggest an association between mean behavior and plasticity, while a correlation between the slopes would imply linked plastic responses to different environmental gradients. Note that individuals may show plastic responses even when the population slope could not be differentiated from 0 (Nussey et al., 2007). We included only one (the first) recording per focal individual into the models (thereby simplifying model structure), because the number of individuals recorded multiple times was relatively low. All three response variables were normally distributed. All continuous variables were *z*‐transformed, as they were measured on different scales. *Z*‐transformation ensured that the mean of the variables was 0, and their standard deviation was 1, maintaining the original direction of the variables.

First, we built a bivariate LMM for SL and MF. We initially aimed to include all three song traits as multiple response variables, but this analysis was not feasible due to the smaller sample size for complexity. In the bivariate model, the following fixed effects were included for both response variables: binary age of the focal individual (1 year old or older) and date of the recording (as control variables), the order of songs within the recording in interaction with context (whether the recording was made after female or male stimulus, or with no stimuli) and singing position. The random part of the model contained year, focal bird identity, a random slope over the order of songs and over the singing positions for focal bird identity with correlations between random intercepts and slopes. The whole random structure for individual identity (intercept, slope for order of songs, slope for singing position) was estimated separately for the three contexts in which the recordings were made, as we expected context‐based patterns based on our previous research (Jablonszky et al., 2021). We also considered correlations between random intercepts and slopes across traits (but only within the contexts).

We built a second, similar, but univariate model for complexity. As in the bivariate model, the fixed variables were age, date, the order of songs in interaction with context, and singing position. The random part was also similar, including intercepts, slopes for the order of songs and singing position for individual identity separately for the contexts, and year‐specific effects.

The identity of the stimulus bird could also influence song (Heinig et al., 2014; Ronald et al., 2015). However, additional analyses incorporating stimulus identity as a random effect suggested negligible effect on our results (Tables S2 and S3), so we present here the results without this term. Context‐dependent response to the singing position could also be predicted. However, we did not introduce more complex models in this direction based on (1) theoretical considerations presented in the Introduction, and (2) the results of additional models with an interaction term between singing position height and context (Table S4). We also conducted additional analyses with at most 30 songs included per individual, as the number of songs available per male greatly varied in our dataset (9–126 songs). The analyses showed the same tendencies as with the full dataset (although some parameters were not significant anymore due to the lower sample sizes), which further proved the robustness of our results (Tables S5 and S6).

#### 2.4.2 Repeatability of song plasticity

We assessed the repeatability of plastic responses (slopes of reaction norms) in song traits within which we had found among‐individual variability in the random slopes. In the repeated measures scenario, we had 77 recordings from 37 birds from different days or years (3394 songs in total). We only used data from birds where the repeated measurements were recorded in the same context (24 out of 37). Repeatability was calculated with a two steps procedure using univariate LMMs including only individuals with repeated song recordings. We used a two‐step procedure instead of more complex models due to our modest sample size in terms of individuals with repeated data. First, we built two models similar to those described above for each song trait–context combination where we had found variation in random slopes, one for the first and one for the second recordings of the same birds. Age, date, the order of songs, and singing position were the fixed effects, while year and identity (intercept and only slopes found to be variable in the previous analyses) were included as random effects. All posterior samples for the slope estimates for each individual were extracted. Second, we built models using individual slope estimates (repeated with all 1000 samples from the posterior of the previous models built for the first and second recordings of the individuals) as the response variable, order of recording as a fixed factor, and individual identity as a random effect. Based on the posteriors of the latter models, we calculated repeatability (as the posterior means of the ratio of among‐individual variance and total variance), and the 95% credible intervals.

#### 2.4.3 Details of the statistical models

We built random regression models using the ‘MCMCglmm’ package (Hadfield, 2010). We defined parameter expanded priors for the random effects (Gelman & Hill, 2007; Moiron et al., 2020; Patrick & Weimerskirch, 2015). We repeated the analyses using priors with the inverse‐Wishart distribution to check if the results depended on the priors. These models yielded somewhat larger variance estimates, but the results were qualitatively similar to those shown here. The models ran for 510,000 iterations. We set a burn‐in of 10,000 samples, which were discarded at the beginning, and a thinning interval of 500. The trace and distribution of all variables, and the autocorrelation between iterations were checked visually. Mixing and convergence were also checked using the Gelman–Rubin statistics (Gelman & Rubin, 1992). We considered an estimate significant if the 95% credible interval excluded 0. Variance estimates are constrained to be positive, so their significance could not be assessed in the above way. Therefore, we also inspected the whole posterior distribution in addition to the credible intervals (see Figure S1). Variance estimates were compared by calculating the difference between the corresponding posteriors and then calculating the 95% credible interval from the posterior distribution of the difference. We also calculated the probability of direction (p_−_ or p_+_), which is the proportion of posterior samples of the median sign (Makowski et al., 2019). The probability of direction was calculated using a function from the ‘bayestestR’ package (Makowski et al., 2019) and can be compared to the frequentist *p*‐value according to the formula $p_{\text{two ‐ sided}}=2*\left(1‐p_{‐/+}\right)$ (Makowski, Ben‐Shachar, Chen, et al., 2019).

All statistical analyses were performed in the R 3.6.1 statistical environment (R Core Team, 2019).

## 3 RESULTS

### 3.1 Population‐level responses in song

**FIGURE 1:**
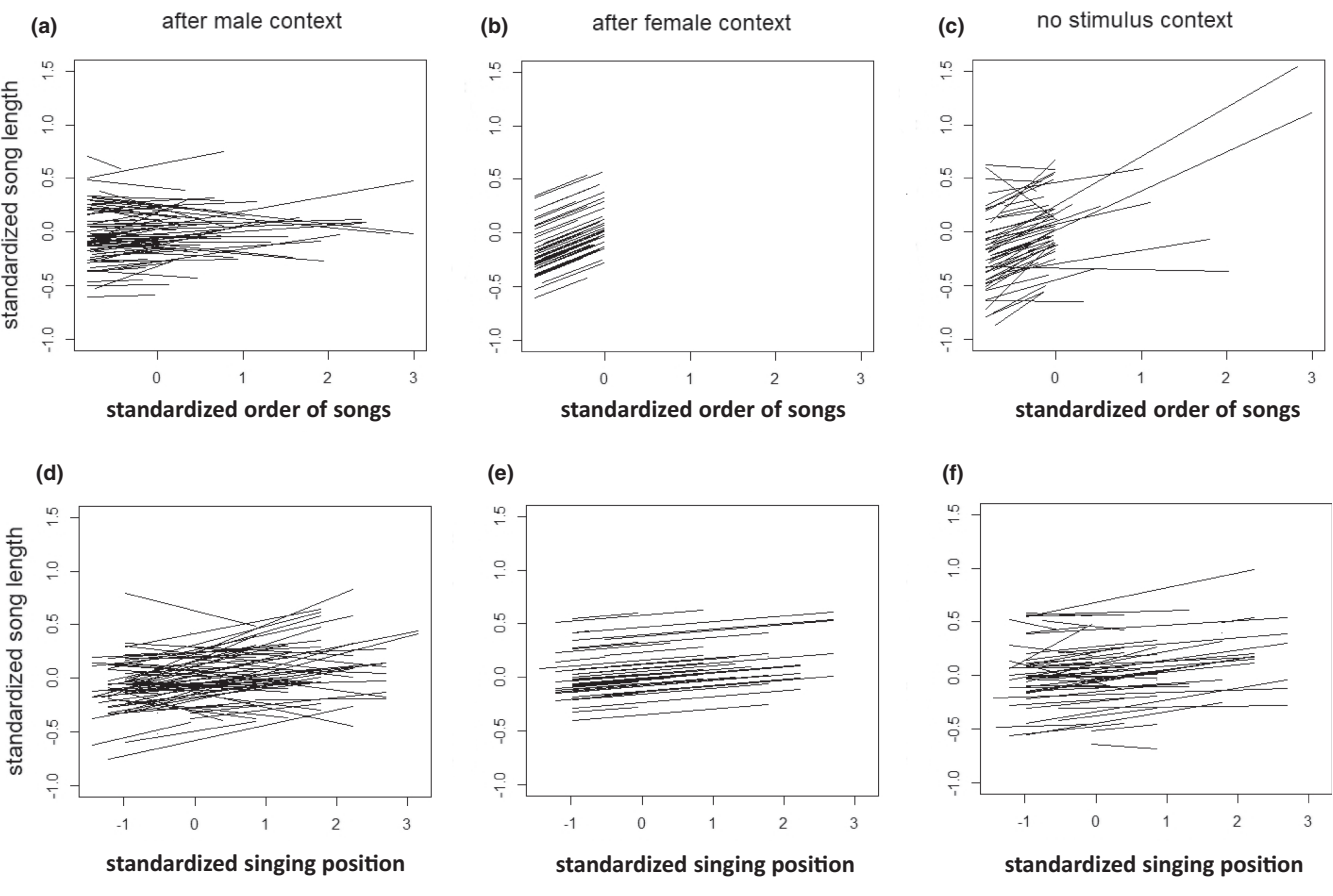
Individual‐specific reaction norms for the order of songs (a–c) and for singing position (d–f) regarding SL. Results are shown for songs after the presentation of male (a, d) and female (b, e) stimuli and for the no stimulus context (c, f), respectively

We found population‐level responses for both the order of songs (in interaction with context) and singing position. The response of SL to the order of songs was significantly different in the female and no stimulus context compared to that in the male context. Specifically, males did not change their song in the male context, but sang longer songs gradually after removing the female stimulus and over time in the no stimulus context (Table 1: Fixed effects, Figure 1a–c). Also, the response of MF to the order of songs in the female and male contexts was significantly different (Table 1). As above, males did not change their song with the order of songs in the male context, but increased MF in the female context. There were significant positive relationships between singing position and both SL and complexity, indicating that birds sing longer and more complex songs from high singing positions (Tables 1 and 2).

**TABLE 1:** Results from the bivariate mixed model investigating among‐individual differences in response to the order of songs and singing position for SL and MF

Fixed effects	Date	Age	Order of songs	Singing position	Context2 (female)	Context3 (no stimuli)	Order:context2	Order:context3
SL	0.052 (−0.028, 0.125)	0.034 (−0.071, 0.145)	0.044 (−0.030, 0.118)	**0.047** (0.009, 0.087)	0.067 (−0.127, 0.257)	0.066 (−0.108, 0.242)	**0.285** (0.035, 0.508)	**0.273** (0.057, 0.517)
MF	0.008 (−0.064, 0.078)	0.011 (−0.091, 0.124)	−0.028 (−0.098, 0.035)	−0.014 (−0.046, 0.021)	0.070 (−0.096, 0.257)	0.014 (−0.141, 0.176)	**0.379** (0.170, 0.586)	0.060 (−0.128, 0.208)

Random effects	SL—intercept	MF—intercept	SL—slope for the order of songs	MF—slope for the order of songs	SL—slope for singing position	MF—slope for singing position
Individual, after male context						
SL—intercept	**0.085** (0.050, 0.125)					
MF—intercept	0.015 (−0.011, 0.048)	**0.100** (0.063, 0.143)				
SL—slope for the order of songs	−0.006 (−0.032, 0.020)	−0.003 (−0.033, 0.026)	**0.041**(0.006, 0.086)			
MF—slope for the order of songs	0.007(−0.018, 0.031)	0.005 (−0.019, 0.035)	0.023(−0.003, 0.053)	**0.034** (0.004, 0.073)		
SL—slope for singing position	0.001(−0.023, 0.023)	0.003 (−0.021, 0.027)	**−0.024** (−0.048, −0.0002)	**−0.023** (−0.048, −0.001)	**0.032** (0.005, 0.062)	
MF—slope for singing position	−0.003 (−0.018, 0.013)	−0.003 (−0.020, 0.011)	−0.011 (−0.030, 0.001)	−0.012 (−0.028, 0.001)	0.014 (−0.001, 0.029)	0.012 (<0.001, 0.026)
Individual, after female context						
SL—intercept	**0.094** (0.038, 0.171)					
MF—intercept	0.026 (−0.019, 0.092)	**0.145** (0.057, 0.229)				
SL—slope for the order of songs	0.011 (−0.031, 0.085)	0.005 (−0.040, 0.083)	0.045 (<0.001, 0.179)			
MF—slope for the order of songs	−0.009 (−0.074, 0.040)	0.006 (−0.051, 0.074)	0.007 (−0.037, 0.059)	0.055 (<0.001, 0.194)		
SL—slope for singing position	0.003 (−0.024, 0.035)	−0.002 (−0.035, 0.029)	−0.006 (−0.038, 0.013)	−0.001 (−0.034, 0.023)	0.018 (<0.001, 0.054)	
MF—slope for singing position	0.001 (−0.019, 0.020)	<0.001 (−0.019, 0.025)	−0.0003 (−0.020, 0.016)	−0.0002 (−0.017, 0.018)	0.002 (−0.009, 0.015)	0.008 (<0.001, 0.030)
Individual, no stimulus context						
SL—intercept	**0.167** (0.071, 0.271)					
MF—intercept	0.004 (−0.059, 0.073)	**0.176** (0.099, 0.268)				
SL—slope for the order of songs	0.053 (−0.032, 0.184)	−0.029 (−0.112, 0.060)	**0.203** (0.016, 0.414)			
MF—slope for the order of songs	−0.012 (−0.044, 0.086)	−0.020 (−0.069, 0.048)	0.013 (−0.070, 0.104)	0.086 (0.004, 0.241)		
SL—slope for singing position	<0.001 (−0.044, 0.042)	−0.022 (−0.062, 0.015)	−0.011 (−0.081, 0.050)	0.004 (−0.035, 0.046)	0.037 (<0.001, 0.090)	
MF—slope for singing position	0.003 (−0.033, 0.037)	−0.010 (−0.044, 0.020)	−0.013 (−0.068, 0.031)	−0.001 (−0.032, 0.034)	0.006 (−0.013, 0.030)	0.024 (<0.001, 0.062)

	SL	MF
Year		
SL	0.020 (<0.001, 0.060)	
MF	0.004 (−0.005,0.021)	0.007 (<0.001, 0.025)
Residual		
SL	**0.653** (0.630, 0.677)	
MF	**0.161** (0.145,0.177)	**0.493** (0.473, 0.512)

Note

β estimates for the fixed effects (population‐level effects) and (co)variances for the random effects with their 95% credible intervals are presented. The reference context was the after male scenario. Random effects variance–covariance matrices are displayed separately as their covariances were allowed to be estimated (individual‐level effects separately for the three social contexts, year, and residual effects). β estimates and covariances for which credible intervals exclude 0, and variances different from 0 based on their credible intervals and posterior distributions, are in bold. Number of songs: 5436, number of individuals: 182.

**TABLE 2:** Results from the univariate mixed model investigating among‐individual differences in response to the order of songs and singing position for complexity

Date	Age	The order of songs (context1)	Singing position	Context2 (female)	Context3 (no stimulus)	Order:context2	Order:context3
*Fixed effects*							
0.043 (−0.014, 0.108)	−0.068 (−0.172, −0.056)	0.024 (−0.115, 0.170)	**0.062** (0.013, 0.108)	0.040 (−0.163, 0.260)	−0.055 (−0.199, 0.078)	0.078 (−0.301, 0.459)	−0.040 (−0.279, 0.161)

	Intercept	Slope for the order of songs	Slope for singing position
*Random effects*			
Individual, after male context			
Intercept	**0.038** (0.010, 0.074)		
Slope for the order of songs	0.011 (−0.018, 0.052)	0.040 (<0.001, 0.135)	
Slope for singing position	−0.010 (−0.027, 0.004)	−0.002 (−0.029, 0.022)	0.017 (<0.001, 0.045)
Individual, after female context			
Intercept	0.029 (<0.001, 0.094)		
Slope for the order of songs	0.007 (−0.033, 0.065)	0.087 (<0.001, 0.278)	
Slope for singing position	−0.0002 (−0.027, 0.021)	0.002 (−0.035, 0.047)	0.023 (<0.001, 0.074)
Individual, no stimulus context			
Intercept	**0.057** (0.010, 0.112)		
Slope for the order of songs	0.002 (−0.039, 0.053)	0.057 (<0.001, 0.186)	
Slope for singing position	−0.003 (−0.025, 0.023)	0.011 (−0.014, 0.050)	0.020 (<0.001, 0.061)
Year	0.005 (<0.001, 0.018)		
Residual	**0.942** (0.901, 0.985)		

Note

β estimates for the fixed effects (population‐level effects) and (co)variances for the random effects with their 95% credible intervals are presented. The reference context was the after male scenario. Random effects variance–covariance matrices are displayed separately as their covariances were allowed to be estimated (individual‐level effects separately for the three social contexts). β estimates and covariances for which credible intervals exclude 0, and variances different from 0 based on their credible intervals and posterior distributions, are in bold. Number of songs: 3848, number of individuals: 155.

### 3.2 Individual‐specific plasticity in song

**FIGURE 2:**
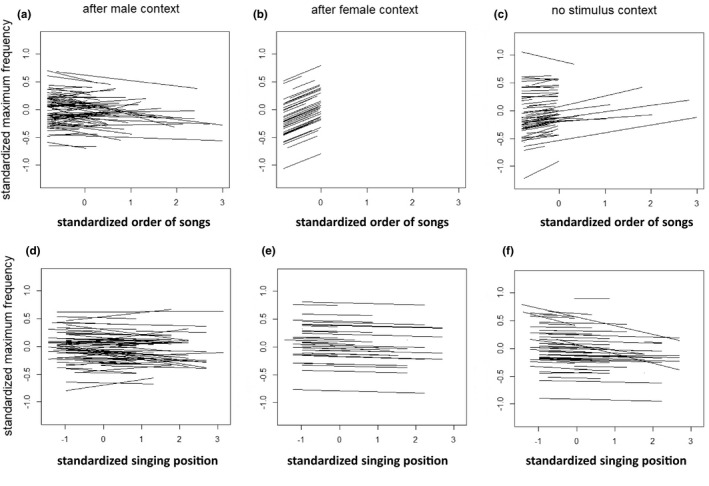
Individual‐specific reaction norms for the order of songs (a–c) and for singing position (d–f) regarding MF. Results are shown for songs after the presentation of male (a, d) and female (b, e) stimuli and for the no stimulus context (c, f), respectively

**FIGURE 3:**
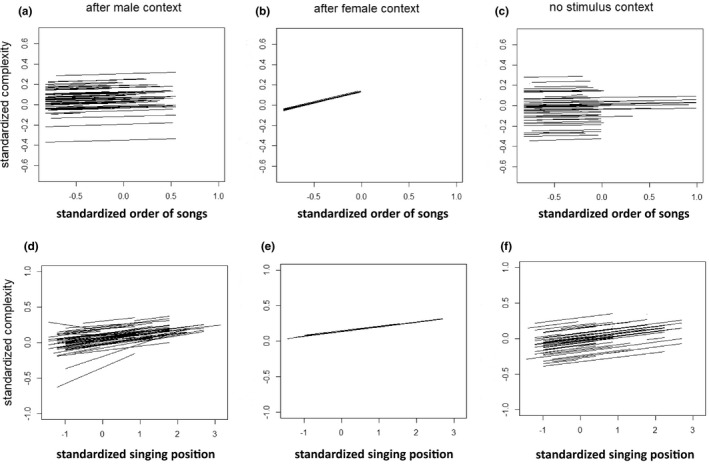
Individual‐specific reaction norms for the order of songs (a–c) and for singing position (d–f) regarding complexity. Results are shown for songs after the presentation of male (a, d) and female (b, e) stimuli and for the no stimulus context (c, f), respectively

Regarding SL, we found among‐individual differences in plasticity for the order of songs in the male stimulus and no stimulus contexts, and for singing position in the male stimulus context (Table 1; Figure 1). Regarding MF, we found among‐individual differences in plasticity for the order of songs in the male stimulus context (Table 1; Figure 2). These significances imply that individuals differ in how plastically they change the length or MF of their songs with respect to the immediate changes in the social environment or in their singing position. For song complexity, we detected no individual differences in plasticity in response to either the order of songs or singing position (Table 2; Figure 3).

We also compared the random intercept and slope variance estimates among contexts and traits (Table S1). We found marginally significant differences (1) between the slopes for the order of songs in the after male stimulus and no stimulus contexts regarding SL, and (2) between the random intercepts of the same contexts regarding MF.

### 3.3 Individual‐level covariances between random terms

In the recordings of males after they were exposed to male stimuli, we found significant covariance (1) between the random slopes for the order of songs and singing position in SL, and (2) between random slopes for singing position in SL and for the order of songs in MF (Table 1). After transformation, the correlations were −0.690 (−0.970, −0.292) *p*_−_ = 99.7% and −0.711 (−0.981, −0.315) *p*_−_ = 99.5%, respectively, showing negative relationships between the plastic responses of the same or different traits to different environmental factors.

### 3.4 Repeatability of song plasticity

We investigated if the slopes of the individual‐specific reaction norms were repeatable for the traits, where individual differences in plasticity were detected. We found moderate repeatability with wide credible intervals in all the random slopes investigated (Table 3). These results confirm that individual‐specific variation in song plasticity in response to both the order of songs and singing position could be repeatable among song recordings made on different days. However, these results must be interpreted with caution, as our sample size for individuals with repeated measurements was relatively low.

**TABLE 3:** Repeatability of random slopes for SL and MF, posterior means with 95% credible intervals are displayed

	SL—the order of songs, after male context	MF—the order of songs, after male context	SL—singing position, after male context	SL—the order of songs, no stimulus context
*N* of songs (*N* of individuals)	785 (11)	785 (11)	785 (11)	263 (6)
Repeatability of random slope	**0.215** (0.0004, 0.703)	**0.264** (0.001, 0.778)	**0.207** (0.0004, 0.708)	**0.326** (0.001, 0.902)

## 4 DISCUSSION

We found population‐level plastic responses as well as among‐individual differences in plasticity in temporal response after exposure to social stimuli and due to the singing position in short song recordings of collared flycatchers. The plastic temporal responses differed across social contexts (male, female stimulus or no stimulus) at both the population and individual levels. Among‐individual differences in plasticity of SL and MF in response to the order of songs or to singing position seemed repeatable in certain contexts, although the estimates had wide credible intervals. In contrast, there was no evidence for consistent variation across recordings for song complexity. Among‐individual variance in plastic responses of song in social contexts may have implications for reproductive success (Schuett et al., 2010). In general, plasticity and repeatable among‐individual variation in plasticity could influence the evolution of behavioral traits (Charmantier & Gienapp, 2014; Dingemanse & Wolf, 2013). Therefore, our results contribute to the understanding of bird song evolution.

Male collared flycatchers consistently differed in the adjustment of their SL and MF regarding the songs recorded after displaying a male stimulus. Considering our experimental setup, we can reasonably assume that the changing social environment caused these individually different plastic responses in the song traits over time, possibly because the exposed listener male represented a key stimulus for the focal males. We have previously observed that individuals adjusted the length and MF of their songs to the identity of listener males (Jablonszky et al., 2021). The results found here show that there are also individual differences in the reaction of males to such social challenges. Individual differences in plasticity after simulated territorial intrusion may arise if lower quality birds have to sing more costly songs to deter other males from their territory than high‐quality males, but they become exhausted in a short time and start singing less costly songs resulting in steeper plasticity slopes. However, plasticity may also reflect cognitive abilities, as individuals that can appropriately perceive rapidly changing social cues may be able to more flexibly adapt to the immediate environment resulting in higher plasticity (Dewitt et al., 1998; Griffin et al., 2015). Individual differences in song plasticity have rarely been investigated, and to the best of our knowledge, the only example comes from ovenbirds (*Seiurus aurocapilla*), for changes in the song types used throughout the breeding season (Thompson et al., 2020). Most studies on animal signal evolution have focused on among‐individual variance, while only a few recent papers, including ours, have examined whether the within‐individual component of trait variation also has evolutionary significance (Campos‐Candela et al., 2019; Hertel et al., 2021; Urszán et al., 2015).

We also found population‐level responses regarding SL and MF in response to the interactive effect of the order of songs and social context. Both SL and MF gradually increased after removing a potential mate, in contrast to the male context. Several studies have shown that birds can alter their song according to the social context (Geberzahn & Aubin, 2014; Gersick & White, 2018; Opaev et al., 2019). Nightingales (*Luscinia megarhynchos*) and zebra finches *(Taeniopygia guttata*), for example, sang shorter songs to females (Glaze & Troyer, 2006; Kipper et al., 2015). In other species, males use different song types in the presence of females or males (Kroodsma et al., 1989; Reichard et al., 2013). Overall, this and previous studies (although the study designs differed) indicate that the ability to adjust the song to the social context is widespread among bird species, but the exact form of this change is species specific. These results, along with the detected among‐individual differences in plasticity in response to the order of songs after a simulated territorial intrusion but not after a simulated female visit, suggest that males react individually to male conspecifics, but show the same, population‐level plastic response to females. Individual differences in plasticity may be adaptive if males benefit from adjusting investment in territory defense (at least regarding SL and MF) to opponent quality (Lattin & Ritchison, 2009; Osiejuk & Jakubowska, 2017). However, differential reactions for females may be costly (e.g., if the chances of pairing is low and/or males have to invest greatly to attract all females). It should be mentioned that the effect of the observer recording the song might have influenced our results regarding plasticity in response to the order of songs, through habituation. However, the individual differences in plasticity after the exposure to male stimuli are unlikely to have emerged due to human presence, given that such among‐individual differences were absent in plasticity in a similar situation after female stimuli.

Population‐level responses were also found for the singing position. Birds generally sang longer and more complex songs from high in the canopy. Singing position height could affect both perceived predation risk, with lower predation risk for birds singing in the dense canopy and sound transmission (Dabelsteen et al., 1993; Gil & Gahr, 2002; Götmark & Post, 1996). However, we cannot determine the cause of the pattern found in our study, as we did not directly assess the predation risk or sound transmission properties at different singing positions. Furthermore, because of the human presence during the song recording, birds may have changed their songs uttered from lower singing positions, because they perceived the experimenter as a potential threat. However, this confounding effect would not alter the study's main conclusions, as we expected changes in song in response to singing position due to parallel changes in predation risk. Changes with singing position height have been shown in the great tit (*Parus major*) for minimum frequency (Bueno‐Enciso et al., 2016) suggesting species‐specific differences in plastic responses to the singing position.

Birds altered their SL differently due to the height of the singing position in the recordings after a male conspecific was displayed. Individual state or quality may be the underlying cause of these individual differences in plasticity in response to the singing position. Males of either higher quality (according to the state‐dependent safety hypothesis; Luttbeg & Sih, 2010) or lower quality (according to the asset protection hypothesis; Wolf et al., 2007) may take more risk when singing, and sing similarly from both exposed and concealed positions. Another possibility is that high quality males can invest more in singing to counteract the effects of sound degradation. However, individual differences in plastic response to the singing position arose only in the male context. Thus, this finding may merely result from the negative covariance with the plastic responses to the order of songs (see below) or the somewhat greater sample size for this social context. These aspects, therefore, warrant further investigations.

We found covariance between the random slopes of different environmental axes and between random slopes of different song traits. The response in SL for changes in social context over time and singing position seemed to be negatively correlated, suggesting trade‐offs between plastic responses to different environmental factors (O'Dea et al., 2022). We also found a significant negative correlation between the plastic responses due to singing position in SL and due to the order of songs in MF, implying that trade‐offs in plasticity may also be present between different behavioral traits.

In summary, we found population‐level responses and individual differences in plasticity in response to different environmental factors in the song of the collared flycatcher. These findings highlight the importance of the social and nonsocial environment in shaping within‐individual variation in some song traits. We also found that among‐individual differences of plasticity were repeatable, which is a rarely investigated issue regarding bird song. Therefore, it might be possible that individual‐specific song plasticity is shaped by selection, and our results may stimulate further studies in this direction.

## CONFLICT OF INTEREST

The authors declare that they have no conflict of interest.

## AUTHOR CONTRIBUTION

**Mónika Jablonszky:** Formal analysis (lead); Investigation (equal); Methodology (lead); Visualization (lead); Writing – original draft (lead). **David Canal:** Investigation (equal); Writing – review & editing (equal). **Gergely Hegyi:** Investigation (equal); Writing – review & editing (equal). **Katalin Krenhardt:** Investigation (equal); Writing – review & editing (equal). **Miklós Laczi:** Investigation (equal); Writing – review & editing (equal). **Gábor Markó:** Investigation (equal); Writing – review & editing (equal). **Gergely Nagy:** Investigation (equal); Writing – review & editing (equal). **Balázs Rosivall:** Investigation (equal); Writing – review & editing (equal). **Eszter Szász:** Investigation (equal); Writing – review & editing (equal). **Sándor Zsebők:** Data curation (equal); Investigation (equal); Resources (equal); Writing – review & editing (equal). **László Zsolt Garamszegi:** Conceptualization (lead); Funding acquisition (lead); Investigation (equal); Supervision (lead); Writing – original draft (supporting); Writing – review & editing (equal).

## Supporting information

Supinfo S1

## Data Availability

The data for this study are deposited in Dryad: https://doi.org/10.5061/dryad.bk3j9kdfc.
