# Supplementary material for: Individual differences in song plasticity in response to social stimuli and singing position
Source: Ecol Evol. 2022 May 2;12(5):e8883. doi: 10.1002/ece3.8883 (PMC9058795; doi:10.1002/ece3.8883)
Supplement: Supplementary file 1 — Supinfo S1 [file ECE3-12-e8883-s001.docx]

Supplementary material for: Individual differences in song plasticity in response to social stimuli and singing position

Figure S1: posterior distribution of the random variances estimated from the bivariate model using song length and maximum frequency (max frequency) as response variables. Results are presented a) for the recordings after male stimulus, b) for the recordings after female stimulus and c) for the no stimulus context


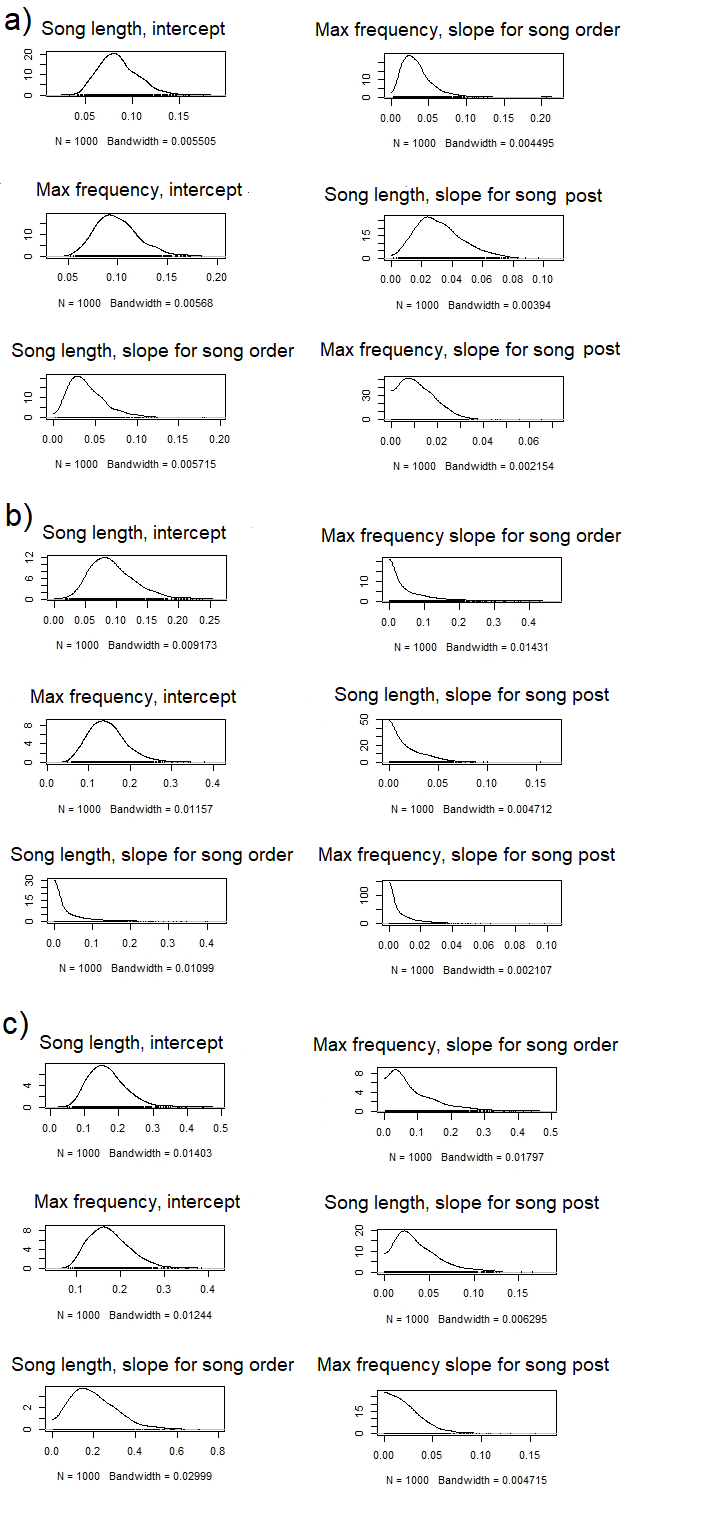


Table S1: Differences in random terms between different contexts and song traits. The mean of the difference of posterior estimates, their 95% credible intervals and probability of direction is displayed. Estimates with probability of direction greater than 95% are in bold

|  | Intercept | Slope for order of songs | Slope for singing position |
| --- | --- | --- | --- |
| Song length |  |  |  |
| After male - after female | -0.009 (-0.095, 0.069) p_-_ = 56.8% | -0.004 (-0.170, 0.087) p_-_ = 63.8% | 0.013 (-0.032, 0.059) p_+_ = 74.4% |
| After male - no stimulus | -0.080 (-0.203, 0.018) p_-_ = 93.5% | **-0.162** (-0.376, 0.032) p_-_ = 95.9% | -0.006 (-0.072, 0.050) p_-_ = 53.4% |
| After female - no stimulus | -0.073 (-0.208, 0.057) p_-_ = 88.3% | -0.158 (-0.459, 0.069) p_-_ = 93.1% | -0.018 (-0.079, 0.041) p_-_ = 73.4% |
| Maximum frequency |  |  |  |
| After male - after female | -0.046 (-0.142, 0.045) p_-_ = 82.3% | -0.021 (-0.179, 0.078) p_-_ = 51.5% | 0.004 (-0.024, 0.027) p_+_ = 68.0% |
| After male - no stimulus | **-0.077** (-0.190, -0.009) p_-_ = 95.1% | -0.052 (-0.217, 0.056) p_-_ = 74.2% | -0.012 (-0.052, 0.024) p_-_ = 70.5% |
| After female - no stimulus | -0.031 (-0.171, 0.086) p_-_ = 69.7% | -0.031 (-0.251, 0.164) p_-_ = 68.4% | -0.015 (-0.062, 0.024) p_-_ = 77.2% |
| Maximum frequency - song length (after male) | 0.015 (-0.049, 0.067) p_+_ = 70.9% | -0.007 (-0.058, 0.037) p_-_ = 61% | -0.020 (-0.051, 0.007) p_-_ = 94% |
| Maximum frequency - song length (after female) | 0.051 (-0.062, 0.156) p_+_ = 82.2% | 0.010 (-0.160, 0.207) p_+_ = 55.6% | -0.011 (-0.052, 0.028) p_-_ = 70.7% |
| Maximum frequency - song length (no stimulus) | 0.009 (-0.150, 0.151) p_+_ = 55.0% | -0.117 (-0.403, 0.160) p_-_ = 83.5% | -0.014 (-0.081, 0.045) p_-_ = 68.4% |

Table S2: Results from the bivariate mixed model investigating among-individual differences in response to order of songs and singing position **for song length (SL) and maximum frequency (MF) with the addition of the random terms male (for context1) and female (for context2) stimulus identity**. β estimates for the fixed effects and (co)variances for the random effects with their 95% credible intervals are presented. The reference context was the after male scenario. Random effects variance-covariance matrices are displayed separately as their covariances were allowed to be estimated (individual-level effects separately for the three social contexts, year and residual effects). β estimates and covariances for which credible intervals exclude 0, and variances different from 0 based on their credible intervals and posterior distributions, are in bold. Number of songs: 5436, number of individuals: 182

| Fixed effects | | | | | | | | | | | | | | | | | | | | | | | | | | | | | | | |  |
| --- | --- | --- | --- | --- | --- | --- | --- | --- | --- | --- | --- | --- | --- | --- | --- | --- | --- | --- | --- | --- | --- | --- | --- | --- | --- | --- | --- | --- | --- | --- | --- | --- |
|  | Date | | | Age | | | | | Order of songs | | | | Singing position | | | | | Context2 (female) | | | | | | Context3 (no stimuli) | | Order:context2 | | | | Order:context3 | | |
| SL | 0.033 (-0.060, 0.114) | | | 0.024 (-0.103, 0.118) | | | | | 0.037 (-0.050, 0.118) | | | | **0.045** (0.004, 0.090) | | | | | 0.077 (-0.126, 0.290) | | | | | | 0.058 (-0.108, 0.254) | | **0.294** (0.061, 0.538) | | | | **0.276** (0.060, 0.534) | | |
| MF | 0.006 (-0.076, 0.081) | | | 0.009 (-0.094, 0.128) | | | | | -0.030 (-0.102, 0.037) | | | | -0.015 (-0.049, 0.022) | | | | | 0.068 (-0.129, 0.283) | | | | | | 0.018 (-0.129, 0.181) | | **0.383** (0.175, 0.621) | | | | 0.064 (-0.104, 0.234) | | |
| Random effects | | | | | | | | | | | | | | | | | | | | | | | | | | | | | | | |  |
| Individual, after male context | | | | | | | | | | | | | | | | | | | | | | | | | | | | | | | |  |
|  | | SL - intercept | | | | | | MF - intercept | | | | SL - slope for order of songs | | | | | MF - slope for order of songs | | | | | | SL - slope for singing position | | | | | | MF - slope for singing position | |  |  |
| SL - intercept | | **0.075** (0.038, 0.113) | | | | | |  | | | |  | | | | |  | | | | | |  | | | | | |  | |  |  |
| MF - intercept | | 0.010 (-0.020, 0.036) | | | | | | **0.095** (0.056, 0.137) | | | |  | | | | |  | | | | | |  | | | | | |  | |  |  |
| SL - slope for order of songs | | -0.007 (-0.038, 0.019) | | | | | | -0.003 (-0.032, 0.023) | | | | **0.038** (0.005, 0.081) | | | | |  | | | | | |  | | | | | |  | |  |  |
| MF - slope for order of songs | | 0.005 (-0.019, 0.029) | | | | | | 0.003 (-0.021, 0.033) | | | | 0.022 (-0.002, 0.054) | | | | | **0.032** (0.002, 0.070) | | | | | |  | | | | | |  | |  |  |
| SL - slope for singing position | | 0.002 (-0.023, 0.025) | | | | | | 0.004 (-0.019, 0.025) | | | | **-0.022** (-0.050, -0.001) | | | | | **-0.022** (-0.044, -0.001) | | | | | | **0.031** (0.005, 0.059) | | | | | |  | |  |  |
| MF - slope for singing position | | -0.002 (-0.016, 0.013) | | | | | | -0.002 (-0.017, 0.013) | | | | -0.010 (-0.028, 0.003) | | | | | -0.011 (-0.027, 0.002) | | | | | | 0.013 (-0.001, 0.030) | | | | | | 0.012 (<0.001, 0.027) | |  |  |
| Individual, after female context | | | | | | | | | | | | | | | | | | | | | | | | | | | | | | | |  |
|  | | | SL - intercept | | | MF - intercept | | | | SL - slope for order of songs | | | | | MF - slope for order of songs | | | | | SL - slope for singing position | | | | | | | MF - slope for singing position | | | |  |  |
| SL - intercept | | | **0.081** (0.017, 0.152) | | |  | | | |  | | | | |  | | | | |  | | | | | | |  | | | |  |  |
| MF - intercept | | | 0.024 (-0.029, 0.079) | | | **0.148** (0.063, 0.238) | | | |  | | | | |  | | | | |  | | | | | | |  | | | |  |  |
| SL - slope for order of songs | | | 0.011 (-0.030, 0.075) | | | 0.006 (-0.042, 0.069) | | | | 0.042 (<0.001, 0.158) | | | | |  | | | | |  | | | | | | |  | | | |  |  |
| MF - slope for order of songs | | | -0.007 (-0.071, 0.047) | | | 0.008 (-0.038, 0.101) | | | | 0.007 (-0.031, 0.057) | | | | | 0.057 (<0.001, 0.190) | | | | |  | | | | | | |  | | | |  |  |
| SL - slope for singing position | | | 0.006 (-0.021, 0.038) | | | -0.001 (-0.037, 0.029) | | | | -0.004 (-0.033, 0.015) | | | | | -0.0004 (-0.034, 0.021) | | | | | 0.018 (<0.001, 0.056) | | | | | | |  | | | |  |  |
| MF - slope for singing position | | | 0.003 (-0.016, 0.022) | | | 0.001 (-0.020, 0.026) | | | | 0.0003 (-0.014, 0.018) | | | | | -0.0002 (-0.020, 0.014) | | | | | 0.002 (-0.006, 0.014) | | | | | | | 0.008 (<0.001, 0.028) | | | |  |  |
| Individual, no stimulus context | | | | | | | | | | | | | | | | | | | | | | | | | | | | | | | |  |
|  | | | SL - intercept | | | | MF - intercept | | | | SL - slope for order of songs | | | | | MF - slope for order of songs | | | | | | SL - slope for singing position | | | | | | MF - slope for singing position | | |  |  |
| SL - intercept | | | **0.151** (0.058, 0.260) | | | |  | | | |  | | | | |  | | | | | |  | | | | | |  | | |  |  |
| MF - intercept | | | -0.003 (-0.058, 0.067) | | | | **0.171** (0.099, 0.266) | | | |  | | | | |  | | | | | |  | | | | | |  | | |  |  |
| SL - slope for order of songs | | | 0.051 (-0.031, 0.171) | | | | -0.033 (-0.128, 0.056) | | | | **0.206** (0.016, 0.439) | | | | |  | | | | | |  | | | | | |  | | |  |  |
| MF - slope for order of songs | | | 0.009 (-0.042, 0.077) | | | | -0.023 (-0.082, 0.033) | | | | 0.013 (-0.067, 0.105) | | | | | 0.082 (0.006, 0.224) | | | | | |  | | | | | |  | | |  |  |
| SL - slope for singing position | | | -0.0004 (-0.046, 0.038) | | | | -0.022 (-0.065, 0.011) | | | | -0.013 (-0.085, 0.042) | | | | | 0.003 (-0.038, 0.040) | | | | | | 0.038 (0.002, 0.090) | | | | | |  | | |  |  |
| MF - slope for singing position | | | 0.004 (-0.029, 0.040) | | | | -0.010 (-0.041, 0.020) | | | | -0.011 (-0.062, 0.030) | | | | | 0.001 (-0.029, 0.031) | | | | | | 0.006 (-0.013, 0.028) | | | | | | 0.022 (<0.001, 0.061) | | |  |  |
| Year | | | | | | | | | | | | | | | | | | | | | | | | | | | | | | | |  |
|  | SL | | | | MF | | | | |  | | | |  | | | | |  | |  | | | |  | | | | | |  |  |
| SL | 0.014 (<0.001, 0.051) | | | |  | | | | |  | | | |  | | | | |  | |  | | | |  | | | | | |  |  |
| MF | 0.002 (-0.006,0.016) | | | | 0.006 (<0.001, 0.024) | | | | |  | | | |  | | | | |  | |  | | | |  | | | | | |  |  |
| Male stimulus ID (context1) | | | | |  | | | | |  | | | |  | | | | |  | |  | | | |  | | | | | |  |  |
|  | SL | | | | MF | | | | |  | | | |  | | | | |  | |  | | | |  | | | | | |  |  |
| SL | 0.017 (<0.001, 0.046) | | | |  | | | | |  | | | |  | | | | |  | |  | | | |  | | | | | |  |  |
| MF | 0.002 (-0.005, 0.013) | | | | 0.004 (<0.001, 0.016) | | | | |  | | | |  | | | | |  | |  | | | |  | | | | | |  |  |
| Female stimulus ID (context2) | | | | | | | | | | | | | | | | | | | | | | | | | | | | | | |  |  |
|  | SL | | | | MF | | | | |  | | | |  | | | | |  | |  | | | |  | | | | | |  |  |
| SL | 0.007 (<0.001, 0.022) | | | |  | | | | |  | | | |  | | | | |  | |  | | | |  | | | | | |  |  |
| MF | 0.002 (-0.003, 0.012) | | | | 0.007 (<0.001, 0.025) | | | | |  | | | |  | | | | |  | |  | | | |  | | | | | |  |  |
| Residual |  | | | |  | | | | |  | | | |  | | | | |  | |  | | | |  | | | | | |  |  |
|  | SL | | | | MF | | | | |  | | | |  | | | | |  | |  | | | |  | | | | | |  |  |
| SL | **0.653** (0.631, 0.681) | | | |  | | | | |  | | | |  | | | | |  | |  | | | |  | | | | | |  |  |
| MF | **0.161** (0.144,0.178) | | | | **0.493** (0.474, 0.512) | | | | |  | | | |  | | | | |  | |  | | | |  | | | | | |  |  |

TableS3: Results from the univariate mixed model investigating among-individual differences in response to order of songs and singing position **for complexity with the addition of the random terms male (for context1) and female (for context2) stimulus identity**. β estimates for the fixed effects and (co)variances for the random effects with their 95% credible intervals are presented. The reference context was the after male scenario. Random effects variance-covariance matrices are displayed separately as their covariances were allowed to be estimated (individual-level effects separately for the two social contexts). β estimates and covariances for which credible intervals exclude 0, and variances different from 0 based on their credible intervals and posterior distributions, are in bold. Number of songs: 3848, number of individuals: 155

| Fixed effects |  | | | |  | |  | | |  | | |  | | | | | |  | | |  | | |  |
| --- | --- | --- | --- | --- | --- | --- | --- | --- | --- | --- | --- | --- | --- | --- | --- | --- | --- | --- | --- | --- | --- | --- | --- | --- | --- |
| Date | | Age | | | | Order of songs | | | | | Singing position | | | Context2 (female) | | | Context3 (no stimulus) | | | Order:context2 | | | Order:context3 | | |
| 0.049 (-0.023, 0.128) | | -0.045 (-0.154, 0.049) | | | | 0.025 (-0.124, 0.183) | | | | | **0.071** (0.023, 0.114) | | | 0.014 (-0.229, 0.243) | | | -0.076 (-0.211, 0.098) | | | 0.064 (-0.304, 0.489) | | | -0.042 (-0.271, 0.201) | | |
| Random effects | | | | | | | | | | | | | | | | | | |  | | |  | | |  |
| Individual, after male context | | | | | | | | | | | | | | | | | | |  | | |  | | |  |
|  | | | Intercept | | | | | Slope for order of songs | | | | Slope for singing position | | | |  |  | | |  | | |  | | |
| Intercept | | | 0.027 (<0.001, 0.056) | | | | |  | | | |  | | | |  |  | | |  | | |  | | |
| Slope for order of songs | | | 0.013 (-0.014, 0.059) | | | | | 0.044 (<0.001, 0.148) | | | |  | | | |  |  | | |  | | |  | | |
| Slope for singing position | | | -0.006 (-0.023, 0.006) | | | | | -0.004 (-0.029, 0.018) | | | | 0.016 (<0.001, 0.042) | | | |  |  | | |  | | |  | | |
| Individual, after female context | | | | | | | | | | | | | | | | | |  | | |  | | |  |  |
|  | | | | Intercept | | | | | Slope for order of songs | | | Slope for singing position | | |  | |  | | |  | | |  | | |
| Intercept | | | | 0.015 (<0.001, 0.055) | | | | |  | | |  | | |  | |  | | |  | | |  | | |
| Slope for order of songs | | | | 0.007 (-0.019, 0.055) | | | | | 0.056 (<0.001, 0.205) | | |  | | |  | |  | | |  | | |  | | |
| Slope for singing position | | | | 0.0003 (-0.017, 0.018) | | | | | 0.002 (-0.029, 0.038) | | | 0.019 (<0.001, 0.065) | | |  | |  | | |  | | |  | | |
| Individual, no stimulus context | | | | | | | | | | | | | | | | | | | |  | | |  | | |
|  | | | | Intercept | | | | | Slope for order of songs | | | Slope for singing position | | |  | |  | | |  | | |  | | |
| Intercept | | | | 0.044 (<0.001, 0.092) | | | | |  | | |  | | |  | |  | | |  | | |  | | |
| Slope for order of songs | | | | 0.003 (-0.033, 0.051) | | | | | 0.058 (<0.001, 0.189) | | |  | | |  | |  | | |  | | |  | | |
| Slope for singing position | | | | -0.001 (-0.024, 0.017) | | | | | 0.008 (-0.014, 0.044) | | | 0.015 (<0.001, 0.049) | | |  | |  | | |  | | |  | | |
| Year | | | | 0.005 (<0.001, 0.019) | | | | |  | | |  | | |  | |  | | |  | | |  | | |
| Male stimulus ID | | | | 0.009 (<0.001, 0.030) | | | | |  | | |  | | |  | |  | | |  | | |  | | |
| Female stimulus ID | | | | 0.019 (<0.001, 0.044) | | | | |  | | |  | | |  | |  | | |  | | |  | | |
| Residual | | | | **0.942** (0.899, 0.984) | | | | |  | | |  | | |  | |  | | |  | | |  | | |

Table S4: fixed effects from the bivariate (for song length and maximum frequency) and univariate (for complexity) models **considering also the interaction between height and social context**. Otherwise the model specification was the same as described in the main text. The reference context was the after male scenario. For the fixed effects β estimates with their 95% credible intervals are presented. β estimates which credible intervals exclude 0, are in bold

| Fixed effect | Song length | Maximum frequency | Complexity |
| --- | --- | --- | --- |
| Date | 0.052 (-0.026, 0.123) | 0.007 (-0.063, 0.081) | 0.042 (-0.020, 0.112) |
| Age | 0.031 (-0.076, 0.134) | 0.011 (-0.104, 0.102) | -0.071 (-0.170, 0.026) |
| Order of songs | 0.035 (-0.046, 0.119) | -0.037 (-0.111, 0.039) | 0.024 (-0.127, 0.156) |
| Singing position | **0.068** (0.002, 0.127) | 0.009 (-0.041, 0.062) | 0.048 (-0.021, 0.114) |
| Context2 (female) | 0.072 (-0.133, 0.276) | 0.071 (-0.104, 0.262) | 0.038 (-0.154, 0.265) |
| Context3 (no stimuli) | 0.068 (-0.099, 0.238) | 0.017 (-0.140, 0.171) | -0.054 (-0.176, 0.097) |
| Order of songs: context2 | **0.295** (0.020, 0.523) | **0.393** (0.175, 0.607) | 0.071 (-0.290, 0.439) |
| Order of songs: context3 | **0.300** (0.039, 0.549) | 0.068 (-0.135, 0.222) | -0.035 (-0.239, 0.186) |
| Singing position: context2 | -0.016 (-0.119, 0.101) | -0.033 (-0.122, 0.048) | 0.030 (-0.101, 0.183) |
| Singing position: context3 | -0.051 (-0.174, 0.055) | -0.059 (-0.147, 0.031) | 0.028 (-0.082, 0.135) |

Table S5: Results from the bivariate mixed model investigating among-individual differences in response to order of songs and singing position **for song length (SL) and maximum frequency (MF)** **with a maximum of 30 songs included for an individual**. β estimates for the fixed effects and (co)variances for the random effects with their 95% credible intervals are presented. The reference context was the after male scenario. Random effects variance-covariance matrices are displayed separately as their covariances were allowed to be estimated (individual-level effects separately for the three social contexts, year and residual effects). β estimates and covariances for which credible intervals exclude 0, and variances different from 0 based on their credible intervals and posterior distributions, are in bold. Number of songs: 3761, number of individuals: 182

| Fixed effects | | | | | | | | | | | | | | | | | | | | | | | | | | | |  |
| --- | --- | --- | --- | --- | --- | --- | --- | --- | --- | --- | --- | --- | --- | --- | --- | --- | --- | --- | --- | --- | --- | --- | --- | --- | --- | --- | --- | --- |
|  | Date | | | Age | | | Order of songs | | | | Singing position | | | | Context2 (female) | | | | | Context3 (no stimulus) | | | | Order:context2 | | Order:context3 | | |
| SL | 0.044 (-0.039, 0.133) | | | 0.020 (-0.102, 0.148) | | | -0.018 (-0.210, 0.157) | | | | **0.058** (0.006, 0.105) | | | | 0.082 (-0.145, 0.287) | | | | | 0.124 (-0.076, 0.320) | | | | **0.336** (0.035, 0.619) | | **0.428** (0.071, 0.687) | | |
| MF | 0.018 (-0.064, 0.091) | | | -0.023 (-0.139, 0.099) | | | -0.109 (-0.267, 0.047) | | | | -0.012 (-0.053, 0.022) | | | | 0.105 (-0.090, 0.286) | | | | | 0.022 (-0.161, 0.188) | | | | **0.456** (0.207, 0.712) | | 0.084 (-0.190, 0.379) | | |
| Random effects | | | | | | | | | | | | | | | | | | | | | | | | | | | |  |
| Individual, after male context | | | | | | | | | | | | | | | | | | | | | | | | | | | |  |
|  | | | SL - intercept | | | MF - intercept | | | SL - slope for order of songs | | | | MF - slope for order of songs | | | | | | SL - slope for singing position | | | | | | MF - slope for singing position | |  |  |
| SL - intercept | | | **0.132** (0.061, 0.215) | | |  | | |  | | | |  | | | | | |  | | | | | |  | |  |  |
| MF - intercept | | | 0.041 (-0.013, 0.102) | | | **0.132** (0.065, 0.210) | | |  | | | |  | | | | | |  | | | | | |  | |  |  |
| SL - slope for order of songs | | | 0.062 (-0.027, 0.178) | | | 0.063 (-0.025, 0.166) | | | **0.185** (0.0001, 0.406) | | | |  | | | | | |  | | | | | |  | |  |  |
| MF - slope for order of songs | | | 0.040 (-0.037, 0.136) | | | 0.073 (-0.013, 0.189) | | | 0.109 (-0.022, 0.251) | | | | 0.176 (<0.001, 0.386) | | | | | |  | | | | | |  | |  |  |
| SL - slope for singing position | | | -0.010 (-0.048, 0.032) | | | -0.015 (-0.058, 0.021) | | | -0.051 (-0.110, 0.001) | | | | -0.046 (-0.105, 0.012) | | | | | | **0.046** (0.008, 0.086) | | | | | |  | |  |  |
| MF - slope for singing position | | | -0.011 (-0.033, 0.011) | | | -0.014 (-0.038, 0.007) | | | -0.026 (-0.068, 0.003) | | | | -0.023 (-0.062, 0.004) | | | | | | 0.013 (-0.002, 0.033) | | | | | | 0.012 (<0.001, 0.027) | |  |  |
| Individual, after female context | | | | | | | | | | | | | | | | | | | | | | | | | | | |  |
|  | | | SL - intercept | | | MF - intercept | | | SL - slope for order of songs | | | | MF - slope for order of songs | | | | | | SL - slope for singing position | | | | | | MF - slope for singing position | |  |  |
| SL - intercept | | | **0.099** (0.037, 0.174) | | |  | | |  | | | |  | | | | | |  | | | | | |  | |  |  |
| MF - intercept | | | 0.026 (-0.031, 0.090) | | | **0.145** (0.067, 0.237) | | |  | | | |  | | | | | |  | | | | | |  | |  |  |
| SL - slope for order of songs | | | 0.015 (-0.034, 0.091) | | | 0.008 (-0.056, 0.069) | | | 0.053 (<0.001, 0.191) | | | |  | | | | | |  | | | | | |  | |  |  |
| MF - slope for order of songs | | | -0.009 (-0.066, 0.045) | | | 0.008 (-0.056, 0.070) | | | 0.006 (-0.031, 0.061) | | | | 0.053 (<0.001, 0.188) | | | | | |  | | | | | |  | |  |  |
| SL - slope for singing position | | | 0.003 (-0.030, 0.037) | | | -0.002 (-0.040, 0.037) | | | -0.008 (-0.046, 0.014) | | | | -0.0004 (-0.039, 0.023) | | | | | | 0.026 (<0.001, 0.067) | | | | | |  | |  |  |
| MF - slope for singing position | | | 0.002 (-0.017, 0.024) | | | 0.001 (-0.020, 0.028) | | | <0.001 (-0.024, 0.015) | | | | -0.0002 (-0.014, 0.020) | | | | | | 0.003 (-0.009, 0.018) | | | | | | 0.008 (<0.001, 0.030) | |  |  |
| Individual, no stimulus context | | | | | | | | | | | | | | | | | | | | | | | | | | | |  |
|  | | | SL - intercept | | | MF - intercept | | | SL - slope for order of songs | | | | MF - slope for order of songs | | | | | | SL - slope for singing position | | | | | | MF - slope for singing position | |  |  |
| SL - intercept | | | **0.179** (0.059, 0.330) | | |  | | |  | | | |  | | | | | |  | | | | | |  | |  |  |
| MF - intercept | | | -0.003 (-0.079, 0.070) | | | **0.156** (0.057, 0.274) | | |  | | | |  | | | | | |  | | | | | |  | |  |  |
| SL - slope for order of songs | | | 0.082 (-0.042, 0.251) | | | -0.026 (-0.146, 0.081) | | | 0.261 (<0.001, 0.617) | | | |  | | | | | |  | | | | | |  | |  |  |
| MF - slope for order of songs | | | 0.025 (-0.080, 0.140) | | | 0.0001 (-0.086, 0.132) | | | 0.033 (-0.112, 0.226) | | | | 0.247 (<0.001, 0.508) | | | | | |  | | | | | |  | |  |  |
| SL - slope for singing position | | | 0.001 (-0.043, 0.048) | | | -0.015 (-0.065, 0.021) | | | -0.017 (-0.105, 0.044) | | | | 0.008 (-0.048, 0.082) | | | | | | 0.041 (<0.001, 0.098) | | | | | |  | |  |  |
| MF - slope for singing position | | | 0.013 (-0.026, 0.055) | | | -0.023 (-0.058, 0.008) | | | -0.011 (-0.077, 0.052) | | | | 0.004 (-0.047, 0.055) | | | | | | 0.011 (-0.010, 0.038) | | | | | | 0.030 (<0.001, 0.065) | |  |  |
| Year | | | | | | | | | | | | | | | | | | | | | | | | | | | |  |
|  | | SL | | | MF | | |  | | | |  | | | |  | |  | | | |  | | | | |  |  |
| SL | | 0.025 (<0.001, 0.067) | | |  | | |  | | | |  | | | |  | |  | | | |  | | | | |  |  |
| MF | | 0.007 (-0.007,0.028) | | | 0.011 (<0.001, 0.039) | | |  | | | |  | | | |  | |  | | | |  | | | | |  |  |
| Residual | | | | | | | | | | | | | | | | | | | | | | | | | | | |  |
|  | | SL | | | MF | | |  | |  | | | |  | | |  | | | |  | |  | | | |  |  |
| SL | | **0.604** (0.578, 0.633) | | |  | | |  | |  | | | |  | | |  | | | |  | |  | | | |  |  |
| MF | | **0.155** (0.135,0.173) | | | **0.490** (0.465, 0.511) | | |  | |  | | | |  | | |  | | | |  | |  | | | |  |  |

Table S6: Results from the univariate mixed model investigating among-individual differences in response to order of songs and singing position **for complexity with a maximum of 30 song included for an individual**. β estimates for the fixed effects and (co)variances for the random effects with their 95% credible intervals are presented. The reference context was the after male scenario. Random effects variance-covariance matrices are displayed separately as their covariances were allowed to be estimated (individual-level effects separately for the two social contexts). β estimates and covariances for which credible intervals exclude 0, and variances different from 0 based on their credible intervals and posterior distributions, are in bold. Number of songs: 3160, number of individuals: 155

| Fixed effects |  | | |  | |  | |  | | | |  | | | | | | |  | | |  | | |  |
| --- | --- | --- | --- | --- | --- | --- | --- | --- | --- | --- | --- | --- | --- | --- | --- | --- | --- | --- | --- | --- | --- | --- | --- | --- | --- |
| Date | | Age | | | Order of songs | | | | Singing position | | | | Context2 (female) | | | | Context3 (no stimulus) | | | Order:context2 | | | Order:context3 | | |
| 0.034 (-0.029, 0.095) | | -0.099 (-0.208, 0.007) | | | 0.056 (-0.206, 0.302) | | | | **0.056** (0.005, 0.112) | | | | 0.020 (-0.208, 0.247) | | | | -0.052 (-0.236, 0.152) | | | 0.033 (-0.393, 0.466) | | | -0.043 (-0.393, 0.342) | | |
| Random effects | | | | | | | | | | | | | | | | | | |  | | |  | | |  |
| Individual, after male context | | | | | | | | | | | | | | | | | | |  | | |  | | |  |
|  | | | Intercept | | | | Slope for order of songs | | | | Slope for singing position | | | | |  |  | | |  | | |  | | |
| Intercept | | | **0.054** (0.004, 0.123) | | | |  | | | |  | | | | |  |  | | |  | | |  | | |
| Slope for order of songs | | | 0.033 (-0.022, 0.140) | | | | 0.079 (<0.001, 0.284) | | | |  | | | | |  |  | | |  | | |  | | |
| Slope for singing position | | | -0.019 (-0.050, 0.005) | | | | -0.008 (-0.052, 0.031) | | | | 0.020 (<0.001, 0.049) | | | | |  |  | | |  | | |  | | |
| Individual, after female context | | | | | | | | | | | | | | | | | |  | | |  | | |  |  |
|  | | | Intercept | | | | Slope for order of songs | | | | Slope for singing position | | | |  | |  | | |  | | |  | | |
| Intercept | | | 0.027 (<0.001, 0.091) | | | |  | | | |  | | | |  | |  | | |  | | |  | | |
| Slope for order of songs | | | 0.007 (-0.025, 0.071) | | | | 0.086 (<0.001, 0.284) | | | |  | | | |  | |  | | |  | | |  | | |
| Slope for singing position | | | 0.001 (-0.024, 0.023) | | | | 0.003 (-0.040, 0.050) | | | | 0.024 (<0.001, 0.081) | | | |  | |  | | |  | | |  | | |
| Individual, no stimulus context | | | | | | | | | | | | | | | | | | | |  | | |  | | |
|  | | | Intercept | | | | Slope for order of songs | | | Slope for singing position | | | |  | | |  | | |  | | |  | | |
| Intercept | | | 0.079 (<0.001, 0.174) | | | |  | | |  | | | |  | | |  | | |  | | |  | | |
| Slope for order of songs | | | 0.062 (-0.035, 0.219) | | | | 0.214 (<0.001, 0.561) | | |  | | | |  | | |  | | |  | | |  | | |
| Slope for singing position | | | 0.004 (-0.036, 0.037) | | | | 0.026 (-0.032, 0.098) | | | 0.026 (<0.001, 0.071) | | | |  | | |  | | |  | | |  | | |
| Year | | | 0.003 (<0.001, 0.014) | | | |  | | |  | | | |  | | |  | | |  | | |  | | |
| Residual | | | 0.926 (0.880, 0.969) | | | |  | | |  | | | |  | | |  | | |  | | |  | | |
